# Virtual hepatectomy and metastasectomy 3D simulation using an automated and semiautomated tool applied to preoperative CT in the treatment of colorectal cancer metastases

**DOI:** 10.1259/bjrcr.20220018

**Published:** 2022-05-12

**Authors:** Giovanna Sawaya Torre, Andressa Inacio Gomes, Marcelo de Melo Viveiros, Raoni Sant Anna, Renata Emy Ogawa, Marcelo Bruno Rezende, Ronaldo Hueb Baroni, Adriano Tachibana

**Affiliations:** 1 Hospital Israelita Albert Einstein, São Paulo, Brazil

## Abstract

Colorectal cancer represents the most common malignancy of the gastrointestinal tract and the second most frequently diagnosed malignancy in adults. The most common site of metastases is the liver and 40% of patients in stage IV have liver only disease.^1^ Hepatic metastases are the major determinants of morbidity and mortality in these patients, with surgery being the treatment of choice or even curative in these cases.^2^ Therefore, aggressive surgeries should be considered in patients with liver only disease. In this context, hepatectomy and metastasectomy have emerged as promising techniques for improving survival in patients with metastatic disease, also providing long-term cure.^3^ The use of liver volumetrics, tridimensional reconstructions with vessel extraction and 3D virtual surgery simulations allows better surgical planning and potentially decrease transfusions, surgery time and complications.^4^ For major hepatectomies (>4 resected segments), surgical planning with computed angiotomography and liver remnant volume calculation potentially increases the safety of surgery. We report a case in which preoperative 3D surgical simulation was crucial for conducting a safe major hepatectomy in a patient with multiple colorectal liver metastases.

## Clinical presentation

A 57-year-old otherwise healthy male patient presented with 5 months history of change in bowel habits and abdominal pain, associated with weight loss of 10 kg. He had family history of colorectal cancer (paternal grandfather).

**Figure 1.**
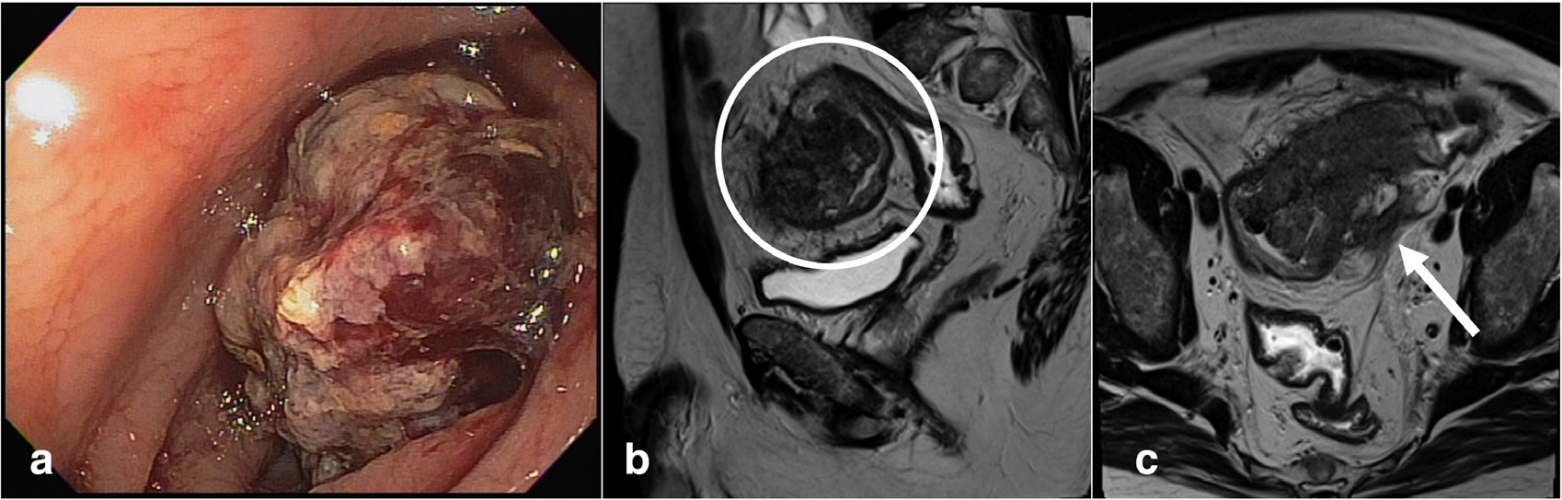
Endoscopic assessment showing vegetative and friable lesion in the distal sigmoid colon (a). Baseline staging MRI showing infiltrative and stenosing lesion of the middle / distal third of the sigmoid in sagittal (b) and axial (c) planes with infiltration of mesosigmoid fat up to peritoneal reflection (arrow).

Then, he went for a colonoscopy which showed a vegetative, friable and obstructive lesion in the distal sigmoid colon, and a biopsy was performed. The anatomopathological study of the biopsy revealed a moderately differentiated tubular adenocarcinoma (Figure 1a).

Magnetic resonance imaging (MRI) performed for staging showed an infiltrative and stenosing lesion of the middle/distal third of the sigmoid colon 8.5 cm long, with signs of transmural extension and infiltration of mesosigmoid fat up to de peritoneal reflection (Figure 1b and c).

**Figure 2.**
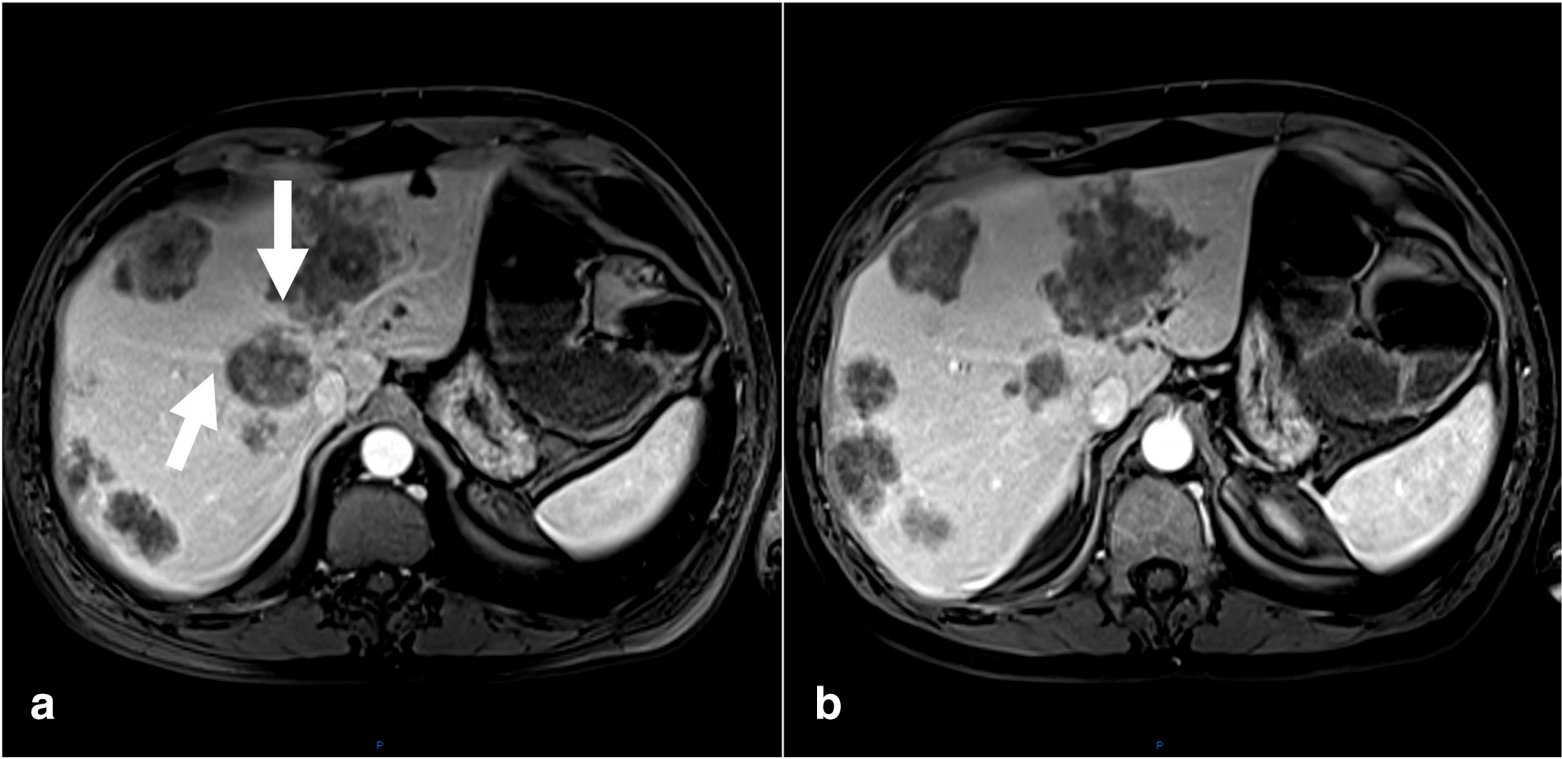
Upper abdomen MR axial *T*_1_-weighted images after intravenous contrast injection show multiple hypovascularised secondary lesions dispersed in both hepatic lobes, including infiltrating the right and middle hepatic veins (arrows).

In the images of the upper abdomen multiple liver metastases were observed affecting right and left hepatic lobes and caudate lobe. Infiltration of the right, left and middle hepatic veins was also noted ^1^ (Figure 2). Carcinoembryonic antigen (CEA) was 121 ng/mL.

18FDG-PET/CT demonstrated high 18FDG uptake in the primary colonic lesion (SUV max = 32.1), regional lymph nodes (SUV max = 26.1) and liver metastases (SUV = 21.4).

A rectosigmoidectomy with colorectal anastomosis was performed. Final anatomopathological diagnosis was invasive adenocarcinoma, without angiolymphatic or perineural invasion, free margins, and no compromised lymph nodes. The surgical staging was pT3N0M1.

Liver lesions were considered inoperable at diagnosis due to invasion of the three hepatic veins. It was chosen to start chemotherapy with FOLFOXIRI (folinic acid, 5-fluorouracil, oxaliplatin and irinotecan) for downstaging and new images were scheduled afterwards to discuss the possibility of hepatic metastasectomy.

After 12 weeks of starting chemotherapy, the CEA was 4,05 ng/mL and a CT and CT-angiography for preoperative planning of liver surgery were performed with the following technical parameters: Canon Aquilion Vision 320 multislice CT scanner with triple-phase contrast acquisition (arterial, portal and equilibrium phases), with intravenous injection of 130 ml of non-ionic iodinated contrast (iobitridol 350 mg/ml) at 4.5 ml/s rate of injection.

### Imaging findings

**Figure 3.**
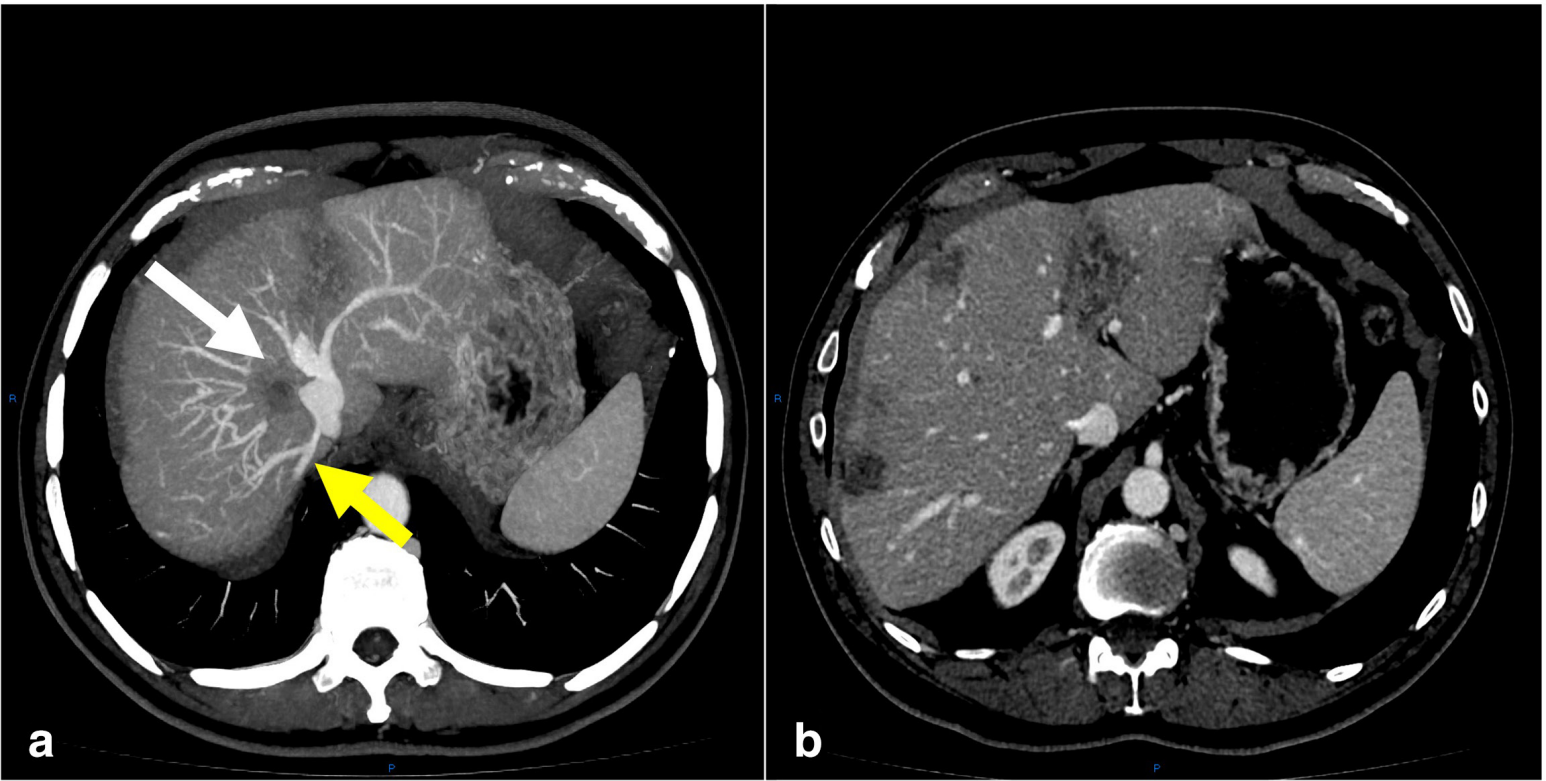
Post-contrast axial sections of restaging abdominal CT. (a) Maximum intensity projection (MIP) showing invasion of the right hepatic vein (white arrow) and accessory hepatic vein (yellow arrow) with important intrahepatic collateral circulation. (b) showing satisfactory result of downstaging, with a smaller number and dimensions of the lesions.

Multiple hepatic metastases were found involving the right and left lobes, but with preserved hepatic artery and portal vein Although there was invasion of the three hepatic veins, two accessory inferior right hepatic veins were observed(Figure 3).

Additionally, collateral veins responsible for draining the territory of the right hepatic vein to the accessory veins were depicted, allowing to continue with the surgical plan. It is worthwhile to mention that resectability depends on the volume of the liver remnant (after neoadjuvant chemotherapy)^2^ ^3^, with sufficient venous and biliary drainage, as well as adequate arterial and portal irrigation. While no data exist regarding the minimum amount of remnant liver following resection, most agree that 25–30 and 40% of the preoperative volume should be preserved for those with normal and abnormal parenchyma (*i.e.,* fibrosis, cirrhosis, steatosis due to preoperative chemotherapy, etc), respectively.^4–6^

**Figure 4.**
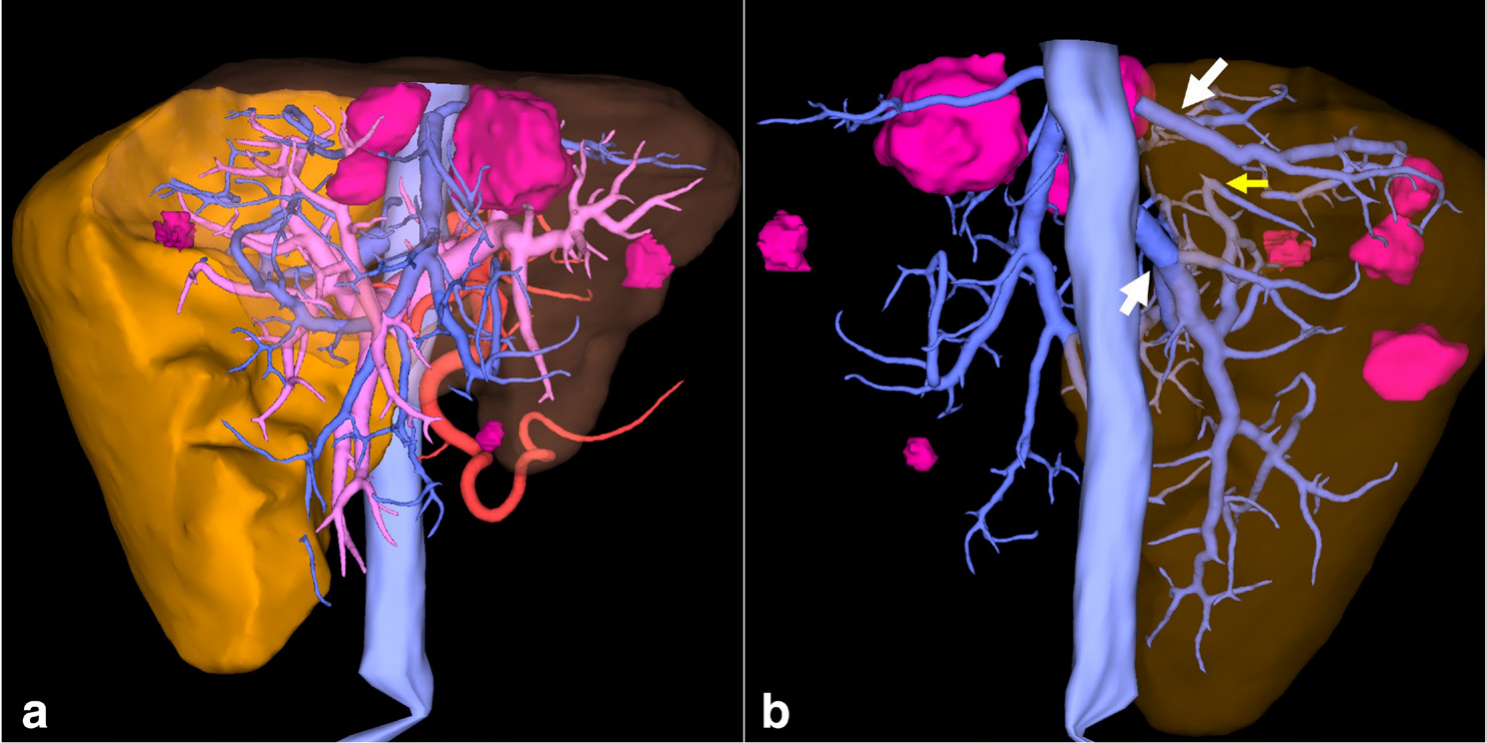
(a) Anterior view of 3D volume rendering image showing virtual hepatectomy with liver remnant estimated in 32.4% (solid yellow) (b) Shows posterior view with remaining liver (transparent brown), hepatic veins and inferior vena cava (light blue), and metastases (pink). Two accessory hepatic veins that drain segments VI and VII (white arrows). Obstructed right hepatic vein also appears (small yellow arrow).

**Figure 5.**
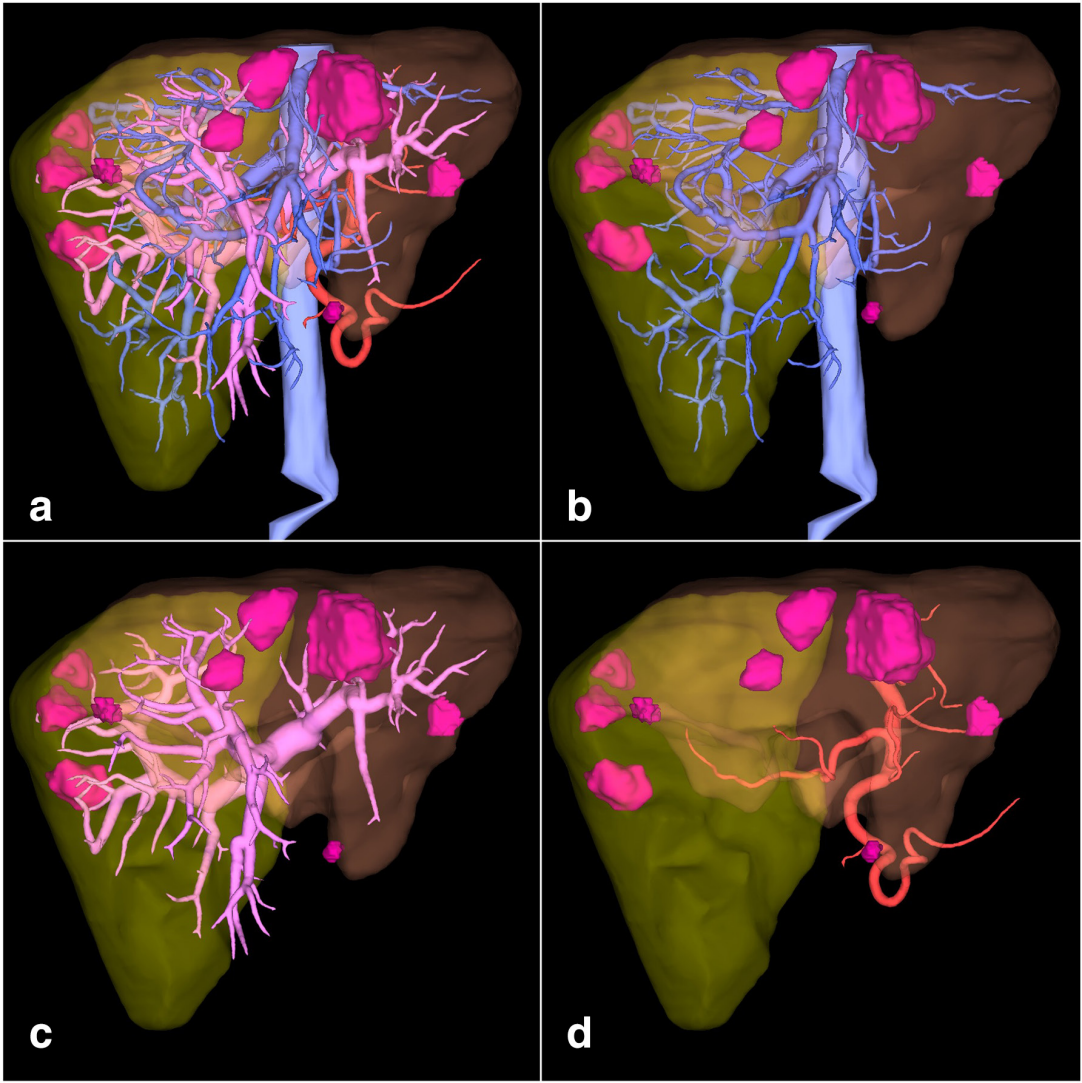
(a) Anterior view of 3D volume rendering images showing virtual hepatectomy with liver remnant (solid yellow), hepatic veins and inferior vena cava (light blue), portal system (light pink), arterial system (red) and metastases (pink). The same reconstruction with (b) just hepatic veins and inferior vena cava, (c) just portal system, (d) just arterial system.

Virtual surgical 3D planning was created using a dedicated system (Synapse 3D, Fujifilm Medical Systems, Tokyo, Japan), which performs automated extraction of the liver, portal veins, inferior vena cava and hepatic arteries and veins and semiautomated segmentations of the liver segments, bile ducts and tumors. Using this tool, it was possible to estimate the tumor volume, simulate the hepatectomy and liver remnant. An extended left hepatectomy and two metastasis resections (metastasectomies) in the segment VII was planned based on the automatic segmentation of the parenchyma perfused by the posterior segmental branch of the right portal vein, with estimated 32,4% of remaining liver (Figures 4 and 5).

## Outcomes

The patient recovered well after surgery, staying in the ICU for 2 days and was discharged from the hospital 5 days after the procedure in good general conditions, with no signs of liver failure or other complications.

A decision was made by the Oncology team not to perform adjuvant chemotherapy. Clinical follow-up 6 months after surgery showed no signs of disease with a serum CEA of 2.16 ng/mL (normal value up to 5.03 ng/mL).

## Discussion

Indications for hepatectomies for colorectal liver metastases are covering more and more patients, regardless of age and number of metastases.^7^ On the other hand, the vascular anatomy of the remnant liver is an important factor in determining resectability and, therefore, an adequate surgical planning becomes decisive.^8^

The surgical plan is based on the technical resectability of the disease to leave a reasonable amount of normal liver after resection, which is an even greater challenge in patients after neoadjuvant chemotherapy, which are often associated with hepatic steatosis.^9–11^ *Maki et al* discuss a new two-stage surgical technique in cases of multiple liver metastases near the venous confluence of the cava using virtual reconstructions in preoperative surgical planning to better assess intrahepatic venous supply and avoid postoperative liver failure.^12^

In conclusion, virtual 3D hepatectomy and metastasectomy using an automated and semiautomated tool have emerged as promising techniques for delivering personalized surgical planning in patients with metastatic disease, potentially improving safety and the chances of a curative surgical treatment.^13^

## Learning points

Virtual 3D hepatectomy and metastasectomy using an automated and semiautomated tool:

- Help define resectability,
- Devise personalised treatment approach,
- Allow better surgical planning, and
- Potentially reduce significant intraoperative complications.

## References

[1] Niekel MC, Bipat S, Stoker J . Diagnostic imaging of colorectal liver metastases with CT, MR imaging, FDG PET, and/or FDG PET/CT: a meta-analysis of prospective studies including patients who have not previously undergone treatment. Radiology 2010; 257: 674–84. doi: 10.1148/radiol.10100729 20829538

[2] Horton KM, Abrams RA, Fishman EK . Spiral CT of colon cancer: imaging features and role in management. Radiographics 2000; 20: 419–30. doi: 10.1148/radiographics.20.2.g00mc14419 10715340

[3] Heymsfield SB, Fulenwider T, Nordlinger B, Barlow R, Sones P, Kutner M . Accurate measurement of liver, kidney, and spleen volume and mass by computerized axial tomography. Ann Intern Med 1979; 90: 185–87. doi: 10.7326/0003-4819-90-2-185 443650

[4] Frankel TL, Gian RK, Jarnagin WR . Preoperative imaging for hepatic resection of colorectal cancer metastasis. J Gastrointest Oncol 2012; 3: 11–18. doi: 10.3978/j.issn.2078-6891.2012.002 22811865 PMC3397633

[5] Chow FCL, Chok KSH . Colorectal liver metastases: an update on multidisciplinary approach. World J Hepatol 2019; 11: 150–72. doi: 10.4254/wjh.v11.i2.150 30820266 PMC6393711

[6] Seyama Y, Kokudo N . Assessment of liver function for safe hepatic resection. Hepatol Res 2009; 39: 107–16. doi: 10.1111/j.1872-034X.2008.00441.x 19208031

[7] Giuliante F, Viganò L, De Rose AM, Mirza DF, Lapointe R, Kaiser G, et al . Liver-first approach for synchronous colorectal metastases: analysis of 7360 patients from the livermetsurvey registry. Ann Surg Oncol 2021; 28: 8198–8208. doi: 10.1245/s10434-021-10220-w 34212254 PMC8590998

[8] Stewart CL, Warner S, Ito K, Raoof M, Wu GX, Kessler J, et al . Cytoreduction for colorectal metastases: liver, lung, peritoneum, lymph nodes, bone, brain. when does it palliate, prolong survival, and potentially cure? Curr Probl Surg 2018; 55: 330–79: S0011-3840(17)30122-3. doi: 10.1067/j.cpsurg.2018.08.004 30526930 PMC6422355

[9] Hazhirkarzar B, Khoshpouri P, Shaghaghi M, Ghasabeh MA, Pawlik TM, Kamel IR . Current state of the art imaging approaches for colorectal liver metastasis. Hepatobiliary Surg Nutr 2020; 9: 35–48. doi: 10.21037/hbsn.2019.05.11 32140477 PMC7026783

[10] Dello SAWG, van Dam RM, Slangen JJG, van de Poll MCG, Bemelmans MHA, Greve JWWM, et al . Liver volumetry plug and play: do it yourself with imagej. World J Surg 2007; 31: 2215–21. doi: 10.1007/s00268-007-9197-x 17726630 PMC2039862

[11] McWhirter D, Kitteringham N, Jones RP, Malik H, Park K, Palmer D . Chemotherapy induced hepatotoxicity in metastatic colorectal cancer: A review of mechanisms and outcomes. Crit Rev Oncol Hematol 2013; 88: 404–15: S1040-8428(13)00114-5. doi: 10.1016/j.critrevonc.2013.05.011 23786843

[12] Maki H, Satou S, Nakajima K, Nagao A, Watanabe K, Satodate H, et al . Two-stage hepatectomy aiming for the development of intrahepatic venous collaterals for multiple colorectal liver metastases. Surg Case Rep 2018; 4: 17. doi: 10.1186/s40792-018-0424-5 29453737 PMC5815977

[13] van der Vorst JR, van Dam RM, van Stiphout RSA, van den Broek MA, Hollander IH, Kessels AGH, et al . Virtual liver resection and volumetric analysis of the future liver remnant using open source image processing software. World J Surg 2010; 34: 2426–33. doi: 10.1007/s00268-010-0663-5 20652701 PMC2936678

